# Supplementary material for: Investigating the Disordered and Membrane-Active Peptide A-Cage-C Using Conformational Ensembles
Source: Molecules. 2021 Jun 12;26(12):3607. doi: 10.3390/molecules26123607 (PMC8231226; doi:10.3390/molecules26123607)
Supplement: Supplementary file 1 [file molecules-26-03607-s001.zip › Supplementary Information For Dobrovolska et al MDPI.pdf]

Supplementary information for:

Investigating the disordered and membrane active peptide A-Cage-C using conformational ensembles

Olena Dobrovolska[1], Øyvind Strømland[1,2], Ørjan Sele Handegård[1], Martin Jakubec[1], Morten L. Govasli[1,3], Åge Skjevik[2], Nils Åge Frøystein[4], Knut Teigen[2] and Øyvind Halskau[1]*

[1]Department of Biological Sciences, University of Bergen, N-5020 Bergen, Norway.

[1]Department of Biomedicine, University of Bergen, N-5020 Bergen, Norway.

[3]Division of Infection and Immunity, University College London, London, United Kingdom.

[4]Department of Chemistry, University of Bergen, N-5020 Bergen, Norway.

*Address correspondence to: Øyvind Halskau, N-5020 Bergen, Norway, Tel: +47-55584563, Fax +47-55589683, E-mail: oyvind.halskau@uib.no

Content description

This document elaborates on the NMR (Table S1, Figure 1S and S2) and statistical methodology used in the work indicated in the title. It also presents some additional results related to the choice of number of clusters that were not presented in the main document (Figure S3), and present representations of all structures within each cluster (Figure S4), as well as results of Molecular Dynamics simulations of A-Cage-C in explicit water (Figure S5).

NMR experiments

The main document covers only basic information regarding the set-up of the ^{31}P, HN-HSQC, HC-HSQC, and triple-resonance experiments. Table S1 includes a more complete set of acquisition parameters. Abbreviations used are Spectral Width (SW), points in the Time Domain (TD), Number of Scans (NS), number of Dummy Scans (DS), and Receiver Gain (RG).

Table S1. Selected acquisition parameters for NMR experiments

<table>
<tr><th rowspan="2">Experiment</th><th colspan="9">Parameter</th></tr>
<tr><th>SW1</th><th>SW2</th><th>SW3</th><th>TD1</th><th>TD2</th><th>TD3</th><th>NS</th><th>DS</th><th>RG</th></tr>
<tr><td>1D ^{31}P</td><td>20 ppm</td><td>-</td><td>-</td><td>32k</td><td>-</td><td>-</td><td>64</td><td rowspan="4">16</td><td>7290</td></tr>
<tr><td>HN-HSQC</td><td>16 ppm</td><td>-</td><td>30 ppm</td><td rowspan="7">2048</td><td>-</td><td>128</td><td>4</td><td>512</td></tr>
<tr><td>HC-HSQC</td><td>13 ppm</td><td>100 ppm</td><td>-</td><td>128</td><td>-</td><td>4</td><td>724</td></tr>
<tr><td>HNCO</td><td rowspan="5">12 ppm</td><td rowspan="5">30 ppm</td><td>20 ppm</td><td>64</td><td rowspan="3">64</td><td>10</td><td>512</td></tr>
<tr><td>HNCA</td><td rowspan="2">30 ppm</td><td>48</td><td>14</td><td rowspan="4">32</td><td>724</td></tr>
<tr><td>HN(CO)CA</td><td>48</td><td>12</td><td>724</td></tr>
<tr><td>CBCANH</td><td rowspan="2">75 ppm</td><td>64</td><td rowspan="2">128</td><td>10</td><td>724</td></tr>
<tr><td>CBCA(CO)NH</td><td>64</td><td>8</td><td>575</td></tr>
</table>

HN-HSQC Fingerprints of A-Cage-C with resonance assignments in the absence of bicelles

The focus of the work is on the membrane associated state and the requirements of binding. To save focus the main document and save space HN-HSQC fingerprints in the absence of bicelles are presented here with resonance assignments superimposed.

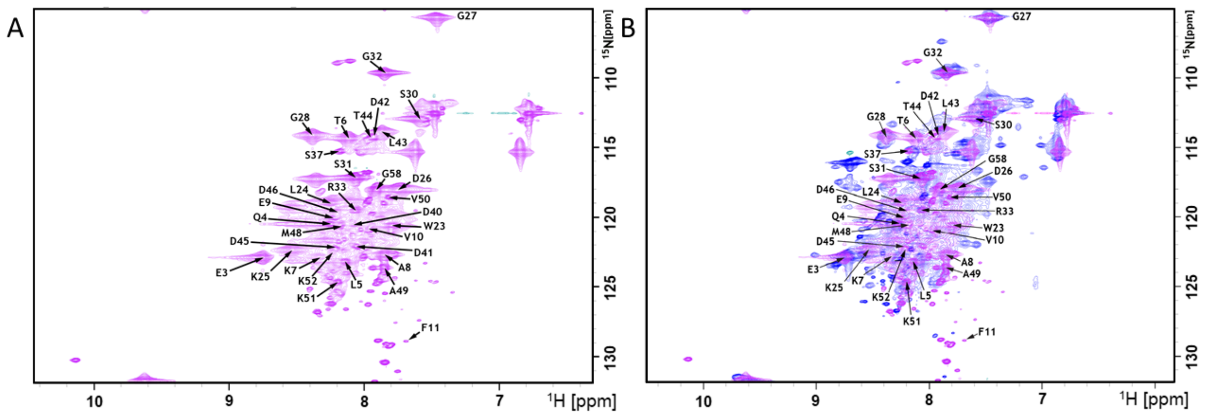

Figure S1: ^{1}H-^{15}N HSQC fingerprints and assignments of A-Cage-C in the absence and presence of bicelles at pH 7.0. A) ^{1}H-^{15}N HSQC of A-Cage-C at pH 7.0 with backbone resonance assignments superimposed. B) As in A, but also the ^{1}H-^{15}N HSQC of A-Cage-C at pH 7.0 in the presence (blue) of bicelles.

Chemical Shift Perturbation analysis of A-Cage-C resonances

This data presents the perturbation in the HN and N resonances of A-Cage-C in the absence of bicelles at pH 7.0 vs pH 4.5.

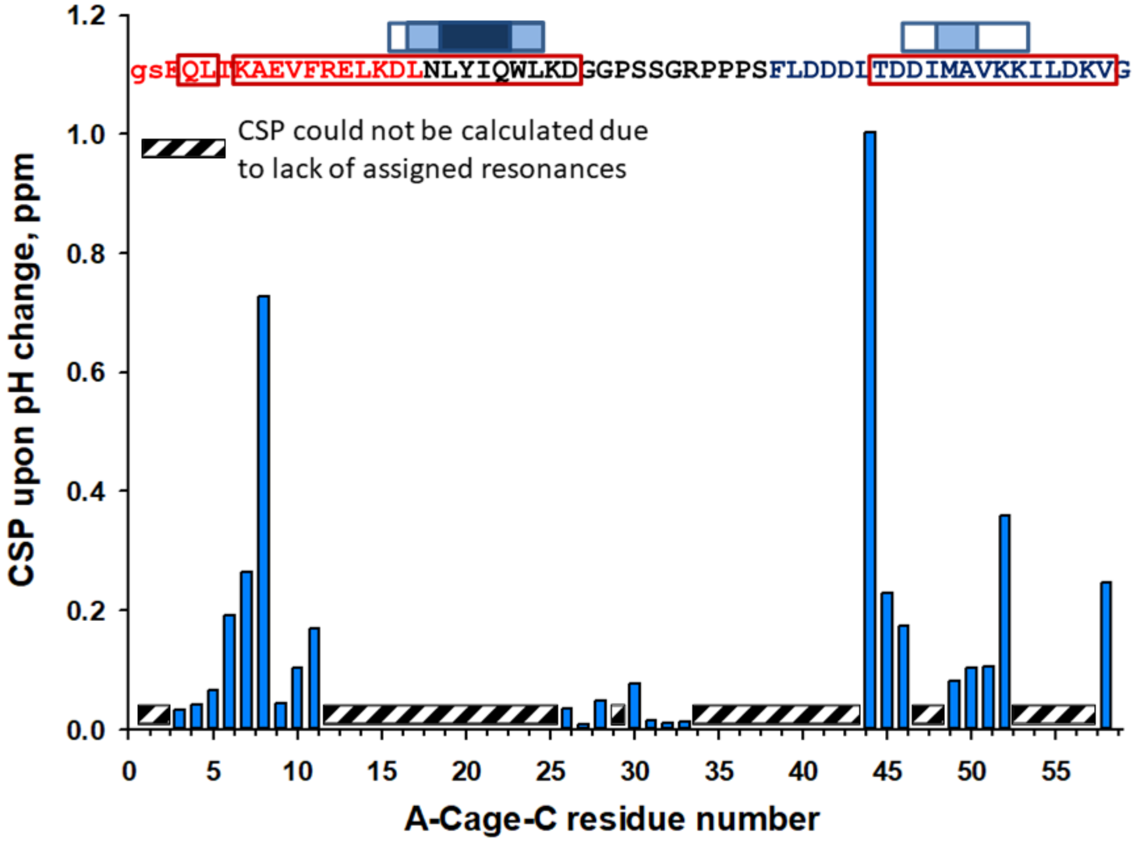

Figure S2: Chemical shift perturbation (CSP) analysis obtained from comparison of the A-Cage-C peptide resonance assignment at pH 7.0 and pH 4.5 in the absence of bicelles. Amino acids at positions indicated by the arrows with stars (*) showed signal broadening beyond detection upon binding to bicelles. Panel insets: A-Cage-C Sequence information aligned with horizontal axis residue number. Red, black and blue capital letters indicate Helix A, Trp-Cage, and Helix C motifs; lower case letters indicate two remaining residues from TEV cleavage; Red frames indicated predicted helixes; White, light blue and dark blue boxes indicate increasing AMYLPRED2 consensus. For further details, see Figure 2 and Materials and Methods in the main document.

Selection of number of clusters

10 000 conformations were generated using Flexible Meccano[1] for A-Cage-C in the presence and absence of bicelles. We used two criteria for selecting the optimal number of clusters to include in our analysis, pseudo F-statistics (pSF) and the Davies-Bouldin index (DBI) [2,3]. The pSF score is a measure of clustering goodness, and compares the backbone mean sum of squares within a cluster, to those between clusters. A cluster selection that provides a high pSF metric is good and indicates that a cluster resembles itself more than it resembles conformations assigned to other groups. Since it uses intermolecular distances it can be viewed as a measure of tightness. The DBI metric is the ratio between in-cluster scatter and between cluster separation. In our case we use distance from centroid structure of each cluster as an indicator of within-cluster scatter, and distance between centroid of each cluster as an indicator of cluster separation. A low DBI metric is indicative of an optimal number of clusters. We present the results of these two criteria in graph form (Fig. S3). The optimal number of clusters for both the absence and presence of bicelles appears to be 2. While the DBI metric for clustering on data acquired in the absence of bicelles (Fig. S3A) suggests that 3 clusters are optimal, going from 2 clusters to 3 cluster creates a cluster with only one conformation in it. pSF indicates 2 clusters in all cases.

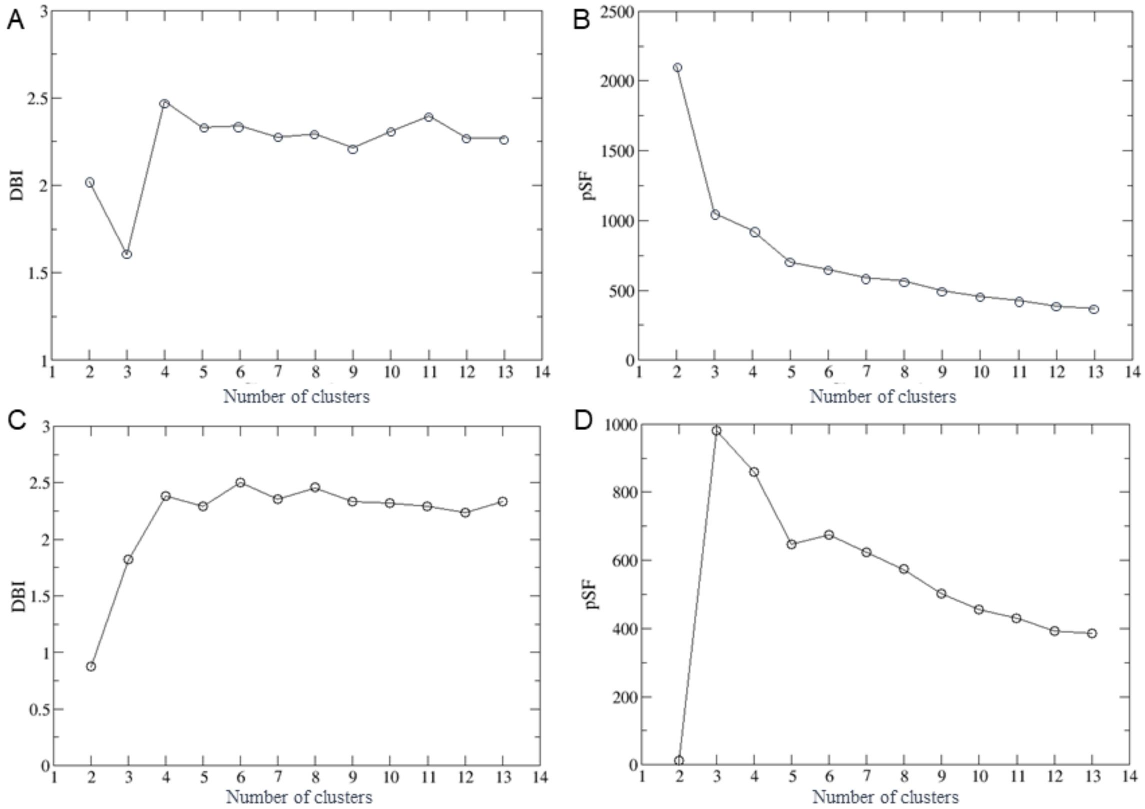

Figure S3: Selection of number of clusters. Clustering statistics for A-Cage-C at pH 4.5 in the absence (A, B) and presence (C, D) of bicelles. The statistical metrics Davies-Boulding index (A, C) and Pseudo F-statistics (B, D) plotted as a function of number of clusters.

Representations of all structures in clusters

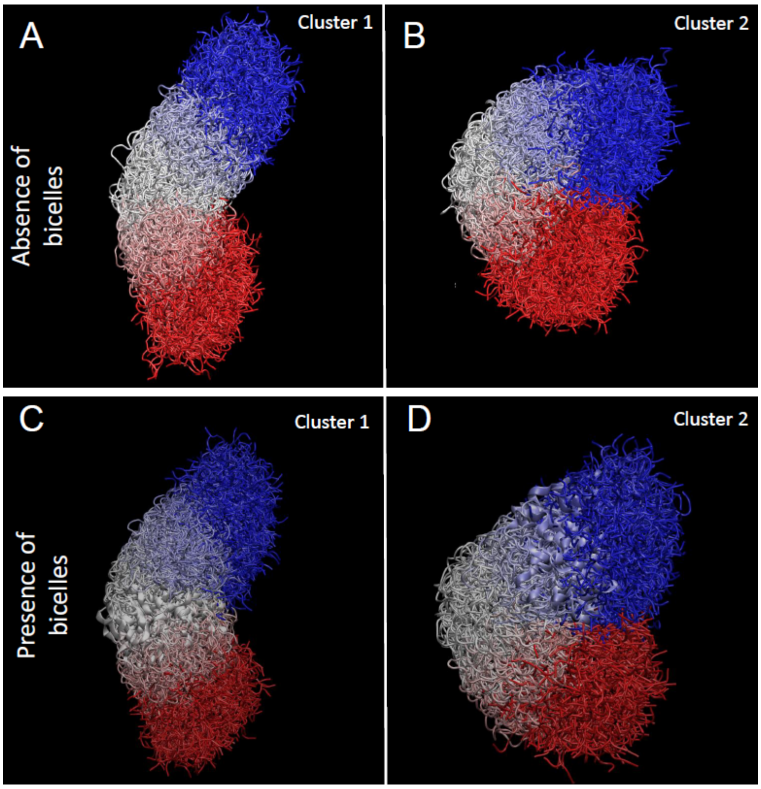

Figure S4: Representations of all structures in clusters. Based on data acquired in the absence and presence of bicelles based on data acquired at pH 4.5. All structural representations have been RMSd-fitted to the backbone atoms of their centroid structure. Colouring is from red (G1) through white to blue (G58). A) Cluster 1 (6349 structures) with no bicelles present. B) Cluster 2 (3651 structures) with no bicelle present. C) Cluster 1 (5105 structures) with bicelles present. B) Cluster 2 (4891 structures) with bicelle present.

MD simulation of A-Cage-C in water

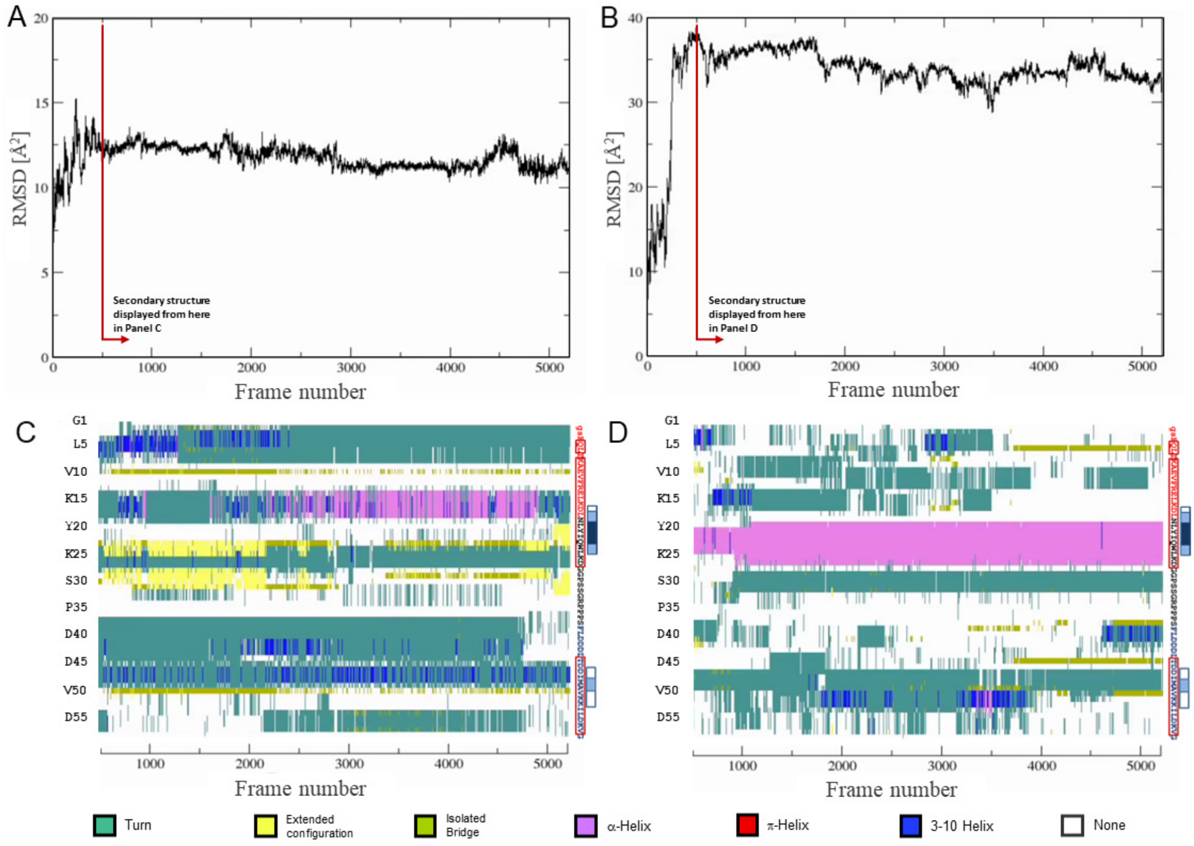

Figure S5: MD simulation of A-Cage-C in water. A) Cα RMSD of compact conformation of A-Cage-C simulated as a function of simulation frame. The RMSD values is calculated as deviation from the starting structure. Each frame of simulation represents a 100 ps, and total simulation time is 520 ns. The red arrow indicates from which frame secondary structure is presented in the panel below. B) Cα RMSD of compact conformation of A-Cage-C simulated as a function of simulation frame. The RMSD values is calculated as deviation from the starting structure. Each frame of simulation represents a 100 ps, and total simulation time is 520 ns. The red arrow indicates from which frame secondary structure is presented in the panel below. C) A-Cage-C compact conformation secondary structure determined by DSSP as a function of simulation time and residue position. Turns, Extended configurations, Isolated bridges, α-Helices, π-Helices, and 3-10 Helices are colour-coded as indicated in at the bottom part of the figure. D) A-Cage-C extended conformation secondary structure determined by DSSP as a function of simulation time and residue position. For the two bottom panels, sequence information aligned with the vertical axis can be found to the right at each panel. Red, black, and blue capital letters indicate Helix A, Trp-Cage, and Helix C motifs; lower case letters indicate two remaining residues from TEV cleavage; Red frames indicate predicted helixes; White, light blue and dark blue boxes indicate increasing AMYLPRED2 consensus. For further details, see Figure 2 and Materials and Methods.

References

1. Ozenne, V. *et al.* Flexible-meccano: A tool for the generation of explicit ensemble descriptions of intrinsically disordered proteins and their associated experimental observables. *Bioinformatics* (2012) doi:10.1093/bioinformatics/bts172.

2. Davies, D. L. & Bouldin, D. W. A cluster separation measure. *IEEE Trans Pattern Anal Mach Intell* **1**, 224–227 (1979).

3. Shao, J., Tanner, S. W., Thompson, N. & Cheatham III, T. E. Clustering Molecular Dynamics Trajectories: 1. Characterizing the Performance of Different Clustering Algorithms. *J Chem Theory Comput* **3**, 2312–2334 (2007).
