# Supplementary figures and images for: Investigating the Disordered and Membrane-Active Peptide A-Cage-C Using Conformational Ensembles
Source: Molecules. 2021 Jun 12;26(12):3607. doi: 10.3390/molecules26123607 (PMC8231226; doi:10.3390/molecules26123607)

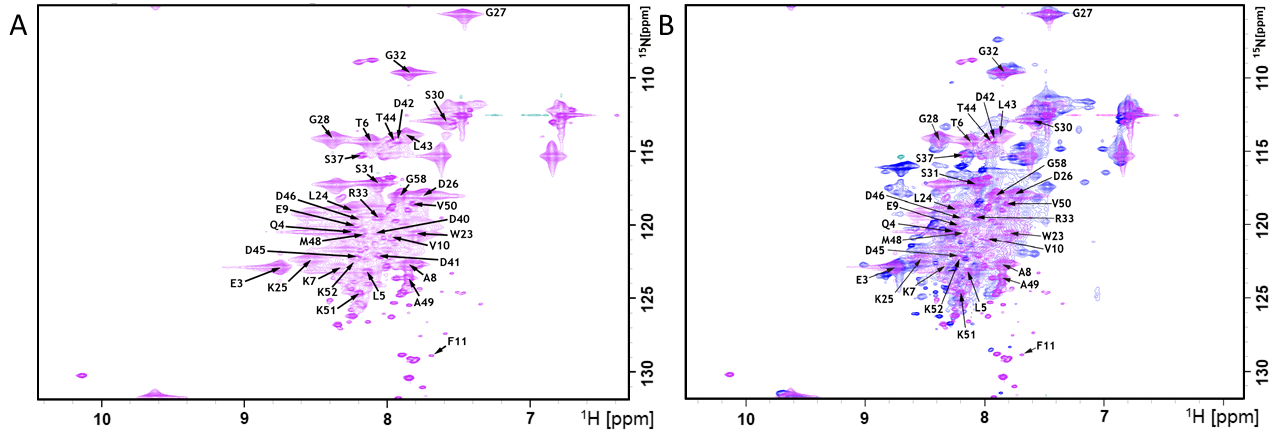

Supplement: Supplementary file 1 [file molecules-26-03607-s001.zip › Figure S1 pH 7 with-without bicelle HSQC.png]

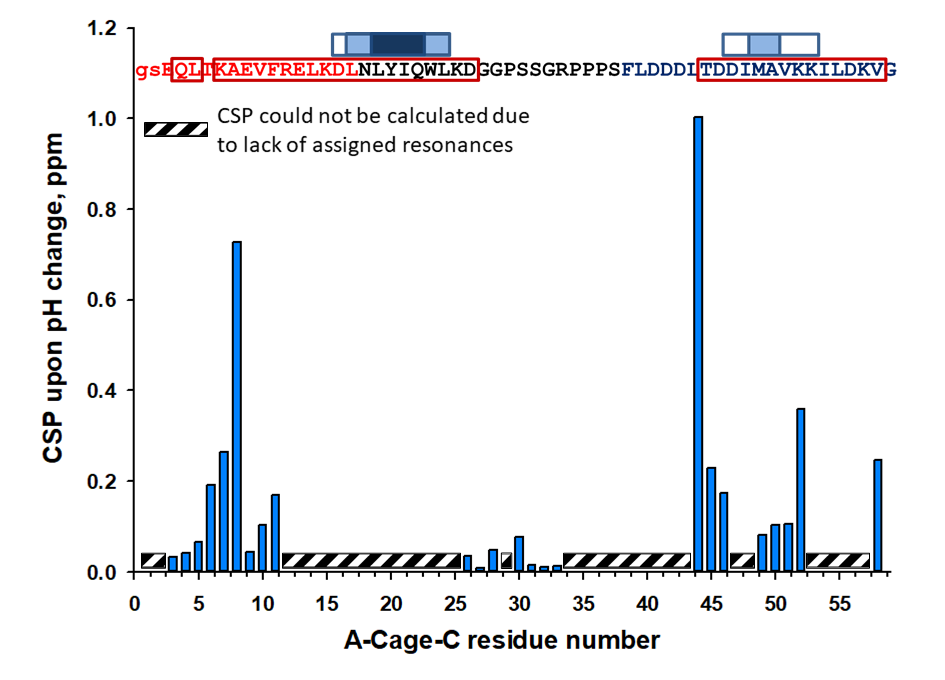

Supplement: Supplementary file 1 [file molecules-26-03607-s001.zip › Figure S2 CSP pH 7 to pH 4.5.png]

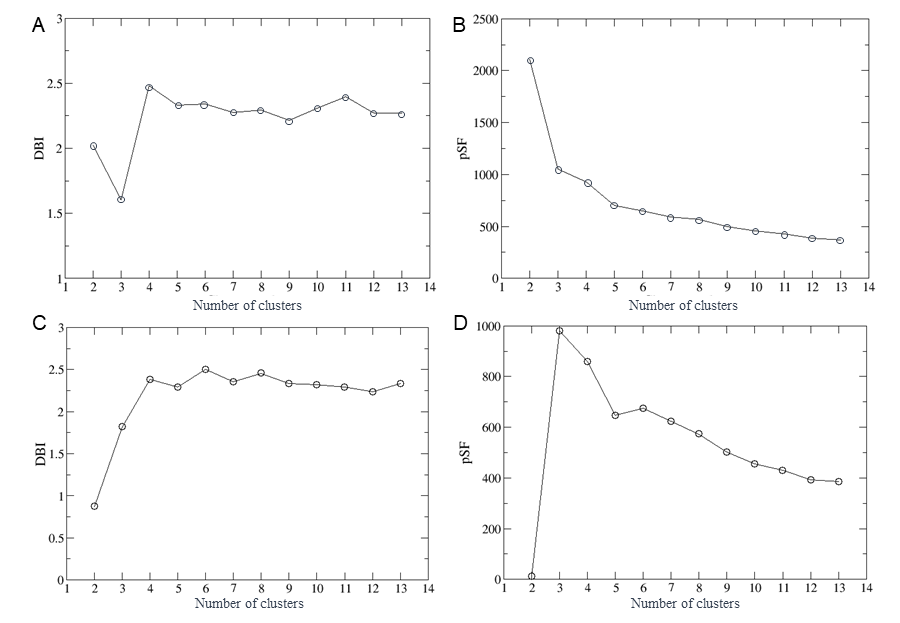

Supplement: Supplementary file 1 [file molecules-26-03607-s001.zip › Figure S3 DBI and pSF.png]

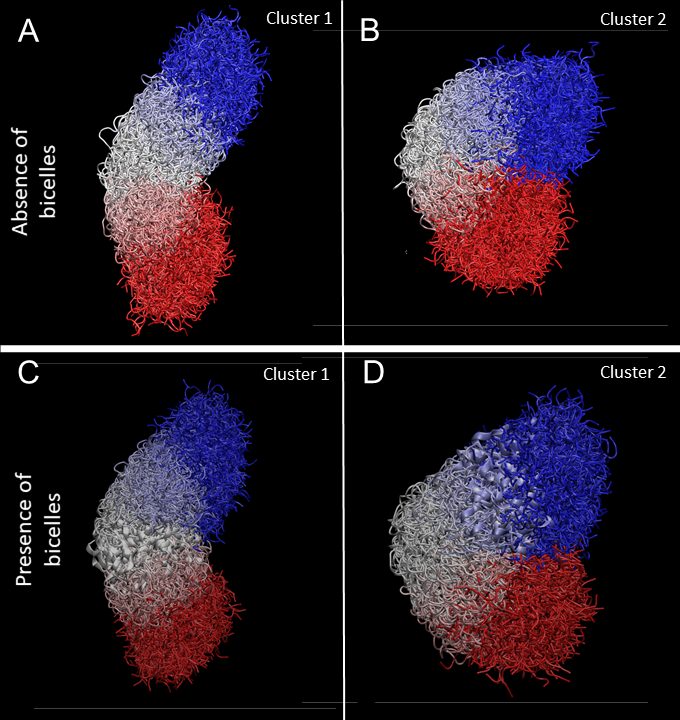

Supplement: Supplementary file 1 [file molecules-26-03607-s001.zip › Figure S4 full clustering absence presence of bicelles.png]

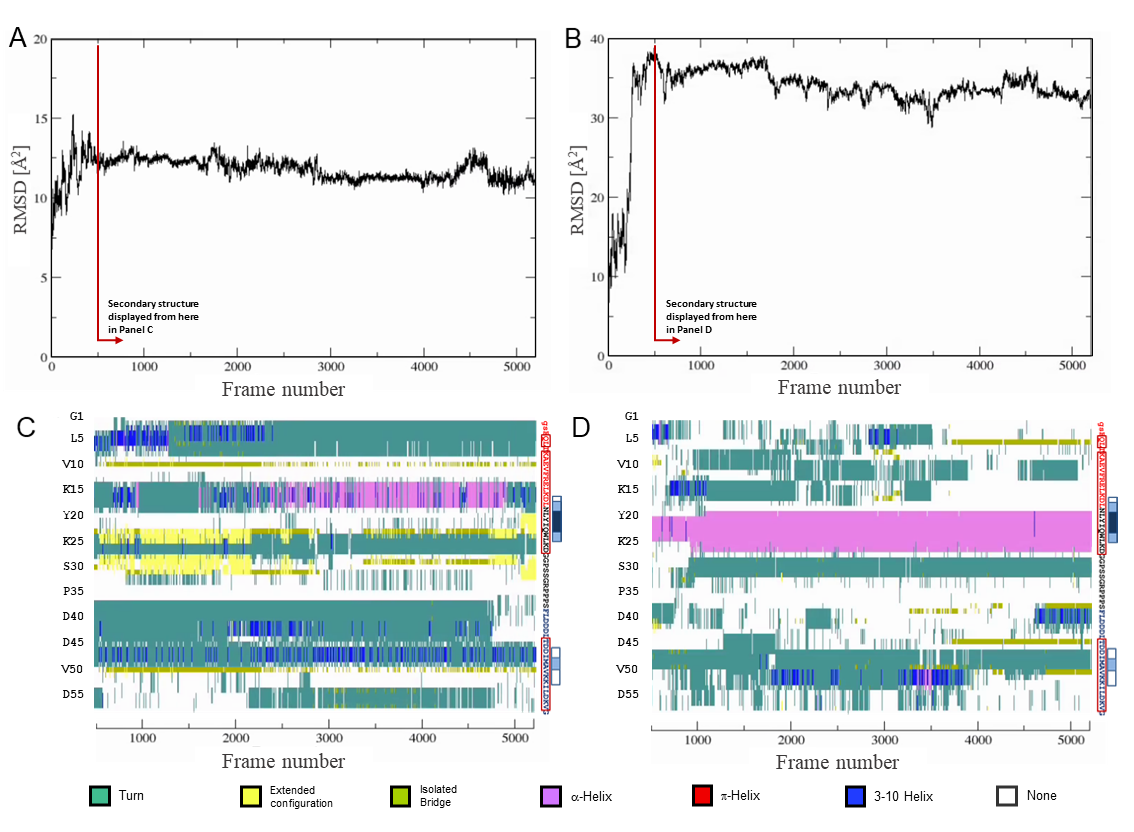

Supplement: Supplementary file 1 [file molecules-26-03607-s001.zip › Figure S5 compt and ext water MD.png]
